# RETRACTION: The Regulation of circRNA RNF13/MiRNA-1224-5p Axis Promotes the Malignant Evolution in Acute Myeloid Leukemia

**DOI:** 10.1155/bmri/9814042

**Published:** 2025-04-29

**Authors:** BioMed Research International

**RETRACTION**: R. Zhang, Y. Li, H. Wang, K. Zhu, and G. Zhang. “The Regulation of CircRNA RNF13/MiRNA-1224-5p Axis Promotes the Malignant Evolution in Acute Myeloid Leukemia,” *BioMed Research International*, no. 2020 (2020): 1–11. https://doi.org/10.1155/2020/5654380.

The above article, published online on 6 October 2020 in Wiley Online Library (wileyonlinelibrary.com), has been retracted by agreement between the Section Editor of the journal Gerald Brandacher; and John Wiley & Sons Ltd.

The retraction has been agreed following an investigation of the concerns raised by *Hoya camphorifolia* on PubPeer [1], which identified an instance of figure duplication.

Specifically:

- Figure 1e: The image of the shRNA-1 expression in HL60 cell colony is identical to the TUSC7 mimic colony of MG63 cells shown in Figure 3c of [2].

As a result of the investigation, the data and conclusions of this article are considered unreliable.

The authors have been informed of the decision to retract the article but did not provide a response.
